# The Evolution Characteristics and Influence Mechanism of Chinese Venture Capital Spatial Agglomeration

**DOI:** 10.3390/ijerph18062974

**Published:** 2021-03-14

**Authors:** Li Yao, Alex Singleton, Pingjun Sun, Guanpeng Dong

**Affiliations:** 1 College of Economics and Management, Southwest University, Chongqing 400700, China; yao66625@163.com (L.Y.); sunpj031@swu.edu.cn (P.S.); 2 Department of Geography and Planning, University of Liverpool, Liverpool L69 7ZT, UK; Alex.Singleton@liverpool.ac.uk; 3 Key Research Institute of Yellow River Civilization and Sustainable Development Collaborative Innovation Center on Yellow River Civilization, Henan University, Kaifeng 475000, China

**Keywords:** venture capital, spatial-temporal distribution, influence mechanism, China

## Abstract

As an emerging financial entity, venture capital has a significant impact on regional development. However, the research on venture capital mainly focuses on the fields of finance, management, and economics, and fewer researchers study venture capital from the perspective of geography and space. This research explored the evolution characteristics and influence mechanism of Chinese venture capital spatial agglomeration. The innovation of this paper lies in including the spatial effect and conducting a spatial econometric analysis of the spatial agglomeration of venture capital in China after the exploratory analysis of the factors affecting the spatial agglomeration of venture capital. Firstly, based on the data of study area, this paper found that the spatial distribution of venture capital in China had an obvious agglomeration characteristic by using multiple measurement methods. Secondly, by constructing the spatial econometric model based on three different spatial weight matrices, we found that the science and technology environment, financial environment, social environment, and entrepreneurial environment levels were the main factors to affect the agglomeration of venture capital. Thirdly, due to the degree of spatial agglomeration of venture capital being divided into three stages in terms of time dimension, after the regression analysis of different periods, we found that the factors which affected spatial agglomeration of venture capital changed significantly with the passage of time. In addition, from the regression results of eastern, central, and western region samples, we can see that the degree of spillover effect was the lowest in the central region, the highest in the western region, and the middle in the eastern region. At last, this paper provided useful policy enlightenment for enterprise innovation, industrial upgrading, and regional economic management.

## 1. Introduction

As an emerging financial entity, venture capital, whose core lies in the value exchange and expansion of capital value across time and space, has a significant impact on regional development and the local production system. Therefore, it has become a hot area of financial geography research [1]. As transregional investment, venture capital influences the economic transition and industrial restructuring in different regions through the way of specialization of capital, shapes a new urban industry and spatial system, and influences the regional, national, and even global financial geographic spatial patterns [2,3]. Therefore, it is a promising scientific subject to study the spatial and temporal distribution and formation mechanism of venture capital institutions. This paper tries to make a useful tentative analysis from this scientific problem.

From the perspective of distribution in the world, venture capital is mainly distributed in the United States, Western Europe, Japan, and some newly industrialized countries and regions. Venture capital originated in the United States in the 1940s and it has successfully supported Intel, Microsoft, Apple Inc. and other international giants [4,5]. The largest concentration of venture capital in America is on the coasts of the Bay Area metropolitan agglomeration, such as San Francisco and San Jose on the West Coast, followed closely by the Boston–New York–Washington line on the East Coast. Japan is the industrially developed country with the highest performance in venture capital investment. Back in the late 1960s, individual Japanese entrepreneurs were involved first in the field of venture capital. Venture capital institutions are mainly concentrated in Osaka, Kyoto, Kobe, Nagoya, Yokohama, and other big cities. In the late 1970s, Britain and Ireland were the first countries in Europe to attract venture capitalists. Actually, Britain, France, and Germany are the three most active economies in the European venture capital industry. Not only developed countries attach great importance to the development of venture capital—in recent years, newly emerging economies have accelerated the development of venture capital and venture enterprises in order to accelerate the development of high-tech industries. They set up venture capital firms with their own money and attract foreign venture capital with preferential policies.

Take China, for example, since the establishment of the first venture capital fund in 1985, China’s venture capital has experienced a short history but rapid development. By the end of December 2018, there have been 24,448 private fund managers registered with the Asset Management Association of China. In recent years, with the rapid development of the venture capital industry in various regions, the venture capital flows to specific regions through the space network, which gradually presents an imbalance in the geographical distribution, and the spatial attribute of this imbalance is inseparable from the regional environment. While, at present, the research on venture capital mainly focuses on the fields of finance, management, and economics, fewer researchers study venture capital from the perspective of geography and space.

Based on the assumption of neoclassical economics, venture capital, as a unique financial capital with strong liquidity in the regional technology innovation network, has the characteristics of nonequilibrium and is concentrated in a few areas, as its core lies in the value exchange and expansion of capital value across time and space. Foreign scholars have paid attention to the imbalance of venture capital spatial distribution earlier. Thompson [6] systematically elaborated the existing venture capital research from the geographical perspective for the first time and emphasized the importance of studying venture capital from the spatial perspective. Most of the early scholars’ research on the spatial layout of venture capital mainly describes the static spatial attributes of venture capital and give some simple explanations from the perspectives of traditional location theory and the operation mechanism of venture capital. They believe that venture capital has the characteristics of spatial agglomeration. In addition, scholars generally believed that venture capital enterprises are mostly concentrated in financial centers, high-tech industrial agglomerations, and regions with a highly successful rate of investment. Since the 1990s, domestic scholars have paid more attention to the research on the spatial distribution of venture capital and made some progress. For example, Cai [7] pointed out that venture capital presented obvious agglomeration characteristics by using the systematic clustering method in China. He [8] believed that venture capital had the nonequilibrium feature of concentrating in a few regions by studying the temporal evolution of spatial agglomeration of venture capital. Wang [9] believed that China has formed an obvious spatial agglomeration venture capital network city, with Beijing and Shanghai as the core nodes. Xu [10] analyzed the venture capital enterprises in Beijing that presented the characteristics of spatial agglomeration and cooperative network. With the change of regional policy, strict regulation of industry standard, time–space compression caused by high-speed rail opening and other influencing factors, the spatial layout of venture capital also changes [11,12,13].

Although the global distribution of venture capital presents a similar trend of spatial differentiation, venture capital activities in different countries also show a unique spatial and temporal attribute. Domestic and foreign studies on the influence mechanism of venture capital spatial distribution mainly focus on the following aspects: (1) Different national and regional environmental factors lead to the spatial differentiation of venture capital, such as technological innovation resources, entrepreneurial atmosphere, financial resources, financial innovation means, etc. [14,15,16,17]. (2) The geographical proximity factors affect the distribution of the venture capital. For example, French [18] discovered the geographic proximity of the investment earlier. Many scholars also have pointed out that it was helpful to reduce transaction costs, resulting in venture capital agglomeration due to the diversity of knowledge and geographical proximity [19,20,21]. (3) Some scholars think that for the reason of reducing the uncertainty and the risk of investment at most, venture enterprises prefer to locate in areas where information is relatively transparent [22,23,24]. (4) Social network connection is also an important factor leading to spatial differentiation of venture capital. For example, Stuart [25] and Fang [26] analyzed the phenomenon of regional agglomeration of venture capital from the perspective of the social network and believed that the social network of venture capitalists would affect the spatial distribution of venture capital. In a word, domestic and foreign researchers attach great importance to the research on the investment environment of venture capital.

On the basis of the above studies, we find that many scholars recognize that venture capital agglomeration is closely related to spatial factors, but few have included the correlation and heterogeneity of spatial dimensions into empirical studies on venture capital agglomeration. In addition, due to the different forms and manifestations of eastern and western economic systems and cities, the mature foreign institutional environment has limited reference significance for the research on the space agglomeration of venture capital in China. Considering China’s huge financial system changes, vast regional space, and large regional development differences, it is of great significance to study the spatiotemporal distribution of venture capital and its expansion mechanism in China. Therefore, it will cover up this very significant spatial difference only if the traditional regression analysis method is used to explain the spatial agglomeration of venture capital. The innovation of this paper lies in including the spatial effect and conducting a spatial econometric analysis of the spatial agglomeration of venture capital in China after the exploratory analysis of the factors affecting the spatial agglomeration of venture capital. By attempting to explore the evolution characteristics and influence mechanism of Chinese venture capital spatial agglomeration, this research extends the study of traditional financial subjects to the emerging financial subject of venture capital. This article aims to expand the research field of financial geography in China and enrich the research theory of financial geography in China and provide references for the formulation of national and regional development policies.

## 2. Materials and Methods

### 2.1. Data Source and Processing

In this study, 31 provincial-level administrative regions in China were included as the research objects (Hong Kong, Macao, and Taiwan were excluded because of incomplete research data). The data of venture capital in this study mainly came from the Zero2IPO Database (a subsidiary of Zero2IPO Group). Zero2IPO Database is the most comprehensive, accurate, and timely professional database. Although the information of the venture capital industry is not completely transparent, and some data cannot be obtained completely, it covers information on investment institutions, funds and managers, mergers and acquisitions, and listing events in China’s venture capital and private equity industries since 1992. At the same time, it also includes the policies and regulations related to venture capital and private equity investment, market development information of various industries, the information of major enterprises, and the corresponding research reports. Other data involved in this paper mainly come from the National Bureau of Statistics, Wind database, the yearbook of science and technology of China, Almanac of China’s Finance, Almanac of Chinese Industrial Economy, and China Statistical Yearbook in years past.

### 2.2. Research Method

#### 2.2.1. Gini Coefficient

Location gini coefficient is a commonly used method to measure industrial agglomeration and geographical concentration. It is often used to measure income inequality and the geographic concentration of production. In this paper, the location gini coefficient is used to evaluate the geographical agglomeration degree of venture capital in various provinces in different periods in China. The calculation formula is:

(1) $$G=\frac{1}{2n^{2}\mu }\underset{j}{\sum }\underset{k}{\sum }\left|S_{j}-S_{k}\right|$$

where *G* is the gini coefficient, $S_{j}$ and $S_{k}$ are the proportion of the venture capital amount of *j* province and *k* province in China, *μ* is the average of venture capital amount in each province, and *n* is the number of provinces. When *G* = 0, it indicates the average distribution of venture capital in each province. When *G* = 1, it indicates that venture capital is concentrated in one province. If an industry is more evenly distributed among regions, its Gini coefficient will be smaller; if an industry is more centrally distributed among regions, its Gini coefficient will be larger.

#### 2.2.2. Concentration Coefficient

Industry concentration coefficient is an important symbol to measure the degree of competition in a certain market. This index represents the market share of the top *n* enterprises in the relevant market of an industry (e.g., number of employees, output value, sales volume, total assets, etc.), which can reflect the concentration level of an industrial market. The calculation formula is:

(2) $$C_{n}=\overset{n}{\underset{i=1}{\sum }}S_{i}$$

In this paper, we modified the index *C_n_* to measure the proportion of the top *n* provinces with the largest venture capital amount, which can also measure the geographical agglomeration degree of the *x* industry in a region [27]. $S_{i}$ is the proportion of the venture capital amount of *i* province. *n* is the number of the selected regions with the largest scale. According to research needs, in industry analysis, *n* is generally taken as 3, 5, and 10.

#### 2.2.3. Location Quotient

The location quotient (LQ) was first proposed by British humanistic geographer Peter Haggett in 1965 and applied in regional analysis [28]. Location quotient index can be used to judge the possibility of industrial agglomeration. In this paper, location quotient coefficient is adopted to evaluate the clustering level of venture capital in research areas in different periods. The calculation formula is:

(3) $$LQ_{i}=\frac{q_{i}}{\sum_{i=1}^{n}q_{i}}/\frac{Q_{i}}{\sum_{i=1}^{n}Q_{i}}$$

where $LQ_{i}$ is location quotient coefficient of venture capital in region *i*. $q_{i}$ represents the amount of the venture capital in region *i*. $Q_{i}$ represents the amount of loans deposited by financial institutions in region *i*. *n* represents the number of study areas. According to the significance of location quotient formula, the critical value of this index is 1. If the regional location quotient coefficient is greater than 1, it means that the professional level of venture capital in this region is higher than the national average level. If the location quotient coefficient is less than 1, it means that the professional level of venture capital in this region is lower than the national average level. The larger the value is, the higher the agglomeration level is, and vice versa.

#### 2.2.4. Spatial Econometric Model

##### Spatial Autocorrelation Test

In order to explore the influencing factors of the spatial agglomeration of venture capital, this paper selected the spatial econometric model method to test. Before the estimation of the spatial econometric model, it is necessary to test the spatial correlation of the spatial agglomeration level of venture capital to determine whether the spatial econometric model is needed.

In this paper, Moran’s I statistic was used to test the spatial correlation. The formula can be expressed as:

(4) $$Moran^{’}I=\frac{\sum_{i=1}^{n}\sum_{j=1}^{n}W_{ij}\left(Y_{i}-\bar{Y}\right)\left(Y_{j}-\bar{Y}\right)}{S^{2}\sum_{i=1}^{n}\sum_{j=1}^{n}W_{ij}}$$

In the formula, $S^{2}=\frac{1}{n}\sum_{i=1}^{n}{\left(Y_{i}-\bar{Y}\right)}^{2}$ and $\bar{Y}=\frac{1}{n}\sum_{i=1}^{n}Y_{i}$. $W_{ij}$ is the spatial weight matrix. $Y_{i}$ represents the observed value of region *i*. *n* is the total number of regions. Moran’s I can be regarded as the product sum of observed values of various regions, whose value range is between −1 and 1. If economic behaviors of various regions are positively correlated in space, its value should be large.

In the selection of spatial weight matrix, adjacent matrix and spatial distance matrix are usually adopted to construct the spatial distance matrix. The geographical factor is not the only factor that determines the spatial correlation, and the geographical distance matrix cannot fully explain the degree of interdependence of spatial clustering of venture capital. Therefore, based on the geographical weight of the economic development level of each region, this paper weighted the proportion of the average per capita GDP. So, the economic space matrix expressed as the product of the geographical distance weight matrix and the diagonal matrix of the proportion of per capita GDP [29]. The specific form of economic distance matrix is:

(5) $$W=w\times diag\left(\frac{\bar{x_{1}}}{\bar{x}},\frac{\bar{x_{2}}}{\bar{x}},\ldots ,\frac{\bar{x_{n}}}{\bar{x}}\right)$$

(6) $$\bar{x_{i}}=\frac{1}{t_{1}-t_{0}+1}\overset{t_{1}}{\underset{t=t_{0}}{\sum }}x_{it},\;\;\bar{x}=\frac{1}{n\left(t_{1}-t_{0}+1\right)}\overset{t_{1}}{\underset{t=t_{0}}{\sum }}\overset{n}{\underset{i=1}{\sum }}x_{it}$$

In the formula, *w* represents the spatial weight matrix of geographic distance. $\bar{x_{i}}=\frac{1}{t_{1}-t_{0}+1}\sum_{t=t_{0}}^{t_{1}}x_{it}$ represents the average level of economic development in region *i* during the study period. $\bar{x}=\frac{1}{n\left(t_{1}-t_{0}+1\right)}\sum_{t=t_{0}}^{t_{1}}\sum_{i=1}^{n}x_{it}$ represents the average level of economic development of all regions during the study period. *t* is the number of time periods, *n* is the number of regions, and *x* is the per capita GDP of regions. Firstly, we needed to normalize the spatial weight matrix so that the sum of the elements in each row of the matrix is 1. 

##### Spatial Durbin Model

The spatial econometric model mainly solves the complex spatial interaction and spatial dependency structure in the regression model. A spatial econometrician holds the opinion that a certain economic geographical phenomenon or an attribute value on a regional spatial unit is related to the same phenomenon or attribute value on a neighboring regional spatial unit [30]. Almost all spatial data have the characteristics of spatial dependence or spatial autocorrelation. The existence of spatial dependence breaks the basic assumption that most classical statistics and econometric analysis are independent of each other. Many scholars have carried out a lot of meaningful discussions on this aspect [31,32,33].

With the development of spatial econometrics, the models and methods based on panel data are becoming more and more mature. In recent years, spatial econometric model has been increasingly used in the research on China’s regional economic development and financial geography [34,35,36,37]. Anselin’s early research on the spatial econometric model was mainly based on cross-sectional data. However, he [38] pointed out that spatial lag term and spatial error term can be included in the Spatial Panel Model when spatial dependence exists among observation individuals, namely the Spatial Lag Model (SLM) and the Spatial Error Model (SEM) based on panel data. Subsequently, LaSage and Pace [39] extended the Spatial Lag Model and proposed the Spatial Durbin Model (SDM). SDM includes both the spatial lag term and the spatial lag term of explained variables. SDM can not only solve the problem of missing variables but also detect the spatial spillover effect of independent variables. Therefore, this paper chose the Spatial Durbin Model for the empirical test. The basic form of SDM is as follows:

(7) $$y_{it}=\rho \overset{N}{\underset{j=1}{\sum }}w_{ij}y_{jt}+x_{it}\beta +\gamma \overset{N}{\underset{j=1}{\sum }}w_{ij}x_{jt}+\mu_{i}+\lambda_{i}+\varepsilon_{it}.\;$$

where $w_{ij}$ is the space weight matrix. $y_{it}$ represents the explained variables of unit *i* in period *t* (i=1,2,…N;t=1,2,…T). ρ is the spatial lag coefficient of the dependent variable. $x_{it}$ represents the explanatory variables of 1×k. β is the estimated coefficient of the explanatory variable. $\sum_{j=1}^{N}w_{ij}y_{jt}$ represents the effect of the explanatory variable element $y_{jt}$ on $y_{it}$ that has a certain spatial position relation with element *i*. γ is the spatial effect coefficient of explanatory variable. $\varepsilon_{it}$ is the random error term and subjects to independent homology distribution. $\mu_{i}$ and $\lambda_{i}$ represent specific effects in space and time. 

#### 2.2.5. Research Hypothesis

The spatial agglomeration of venture capital in China presents a certain temporal-spatial dynamic characteristic. But what causes this phenomenon? What factors affect venture capital in different provinces in China? Since the venture capital market of China is quite different from that of developed countries in Europe and America, this paper proposes the hypothesis of the driving factors of the spatial agglomeration of venture capital in combination with the particularity of venture capital in China. Especially based on the theory of venture capital investment environment system and comprehensive reference factors classification framework, we propose the following hypothesis:

**Hypothesis** **1** **(H1).** *Factor of economic environment: The level of economic environment affects the agglomeration degree of venture capital. The region with superior economic environment conditions can drive the agglomeration degree of venture capital. A good economic environment will create more entrepreneurial opportunities and incubate more start-ups, thus increasing the demand for venture capital [40].*

Empirical research shows that the amount of venture capital activities will fluctuate with the cyclical change of regional macroeconomy. For example, Shachmurove [41] took the venture capital market of the United States as the research object and used real GDP to measure its regional economic environment. The research showed that there is a significant positive correlation between the intensity of venture capital activity and the economic cycle.

**Hypothesis** **2** **(H2).** *Factor of science and technology environment: Science and technology innovation resource advantage has the attractive effect to the venture capital. The relationship between venture capital and technological innovation is a hot research issue. Many scholars believe that venture capital provides enterprises with necessary funds and management experience for research and development, and greatly improves the innovation efficiency of enterprises. These potential opportunities will eventually be explored and converted into entrepreneurial activities, thus increasing the demand for venture capital.*

At the same time, some scholars put forward that technological innovation activities can also stimulate the development of venture capital industry. Kortum [42] and Hirukawa [43] pointed out that there is a large number of technological opportunities in technologically developed regions.

**Hypothesis** **3** **(H3).** *Factor of financial environment: Regions with sound financial market institutions can attract more venture capital. Venture capital is the product of financial innovation, and a sound financial capital market system is the prerequisite for its financing, investment, management, and exit.*

Gompers [44] found that venture investors respond to signals from the securities capital market and increase their investment activities when the signals are good. The financial capital market is the core of the financial market system.

**Hypothesis** **4** **(H4).** *Factor of social environment: A good social environment has an important driving effect on the flow of venture capital and drive venture capital institutions to settle in. Venture capital is a kind of relational investment, and the social network has an important influence on its capital spatial flow. On the one hand, as the medium of information transmission and the source of project transaction, the social network restricts the partner selection and geographical radiation range of joint venture capital [45,46]. On the other hand, the level of social service is a key factor in the location decision of venture capital. The perfect social services and security system provide risk pooling for entrepreneurs, thus stimulating the effective demand for venture capital.*

**Hypothesis** **5** **(H5).** *Factor of entrepreneurial environment: The quantity and quality of entrepreneurial companies affect the location choice of venture capital, and the region with a good entrepreneurial environment can drive the agglomeration degree of venture capital. Entrepreneurial companies are the capital circulation carrier of venture capital, and venture capital is one source of production factors of entrepreneurial companies. There is a synergistic relationship between them.*

When Groh [47] compiled the regional attractiveness index of European venture capital and private equity investment, he pointed out that the rigidity of the labor market and the activity degree of entrepreneurial enterprises have obvious influences on venture capital activities.

#### 2.2.6. Index Selection

##### Dependent Variable

Much research uses the amount of venture capital or the number of events to measure the intensity of regional venture capital activities. Considering that the information of the venture capital industry is not completely transparent, the disclosed information is still representative. Therefore, this study uses the locational quotient coefficient calculated by the amount of venture capital to measure the degree of the concentration of venture capital in the research area (as shown in Table 1).The major data source for studying the venture capital is Zero2IPO Database.

##### Explaining Variable

Generally speaking, in discussing the factor of economic environment, the most commonly used indicator to judge the quality of regional macroeconomic environment is the per capita GDP. Per capita GDP is the GDP (the total market value of all final goods and services produced in the economy of a country or region during a certain period of time) divided by the total population during the period. In this study, the indicator of regional per capita GDP (*Eco*) after adjusting for inflation was selected to measure the factor of economic environment [48,49] (as shown in Table 1). 

To analyze the factor of science and technology environment, we used the indicator of the number of patents in each region (including invention, appearance design, and utility model patents) (*Tec*) (as shown in Table 1). A patent is the product of technological innovation and scientific and technological invention, which is most closely related to innovation activities. Among the numerous scientific and technological outputs, the ownership of a patent is most representative of regional scientific and technological innovation ability. Wong et al. made a systematic and in-depth study on technological innovation capability and related issues from the perspective of patent information analysis [50].

In addition, the indicator of total deposits and loans of financial institutions at the end of the year (*Fin*) is selected to measuring the factor of financial environment (as shown in Table 1). Deposits and loans are the most important sources and ways for financial market organization to provide financial support for economic development. It is generally believed that the basic function of a financial market organization is to optimize the allocation of funds and actively mobilize all economic departments to make contributions to regional economic development.

In this study, we selected two indicators to measure the factor of social environment: one was regional per capita transportation and communication expenses (*Com*), and the other one was proportion of tertiary industry (*Ser*) (as shown in Table 1).

Finally, two variables, entrepreneurial activity and labor market rigidity, were selected to measure regional entrepreneurial environment. The variable of entrepreneurial activity was measured by the number of people aged 15–60 in private companies in the region (*Ent*), while the variable of labor market rigidity was measured by the proportion of state-owned employment in the region (*Lab*) (as shown in Table 1).

## 3. Spatial and Temporal Pattern and Agglomeration Characteristics of Venture Capital

### 3.1. Changes in Spatial Agglomeration Pattern

**Figure 1:**
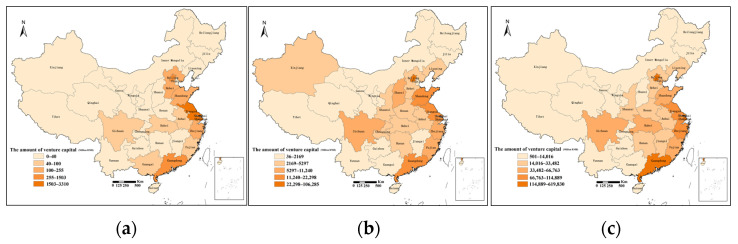
Spatial segmentation map of China venture capital in 2003 (**a**), 2010 (**b**), and 2018 (**c**).

From the perspective of provincial scale, the spatial distribution of venture capital presents significant clustering characteristics in China. As shown in Figure 1, the provinces with the largest amount of venture capital in China are Beijing, Shanghai, Guangdong, Zhejiang, and Jiangsu. For example, in 2018, these five provinces accounted for 86.47% of the country’s venture capital. This fully reflects the high spatial agglomeration of China’s venture capital on the provincial scale, which mainly manifested in first-tier cities and eastern coastal areas. Combined with the spatial distribution of venture capital institutions, venture capital projects also showed significant clustering characteristics, and the two were very similar in spatial form, which indicates that venture capital had local investment preference, and also proves that venture capital had obvious spatial proximity.

### 3.2. Changes in the Degree of Spatial Agglomeration

**Figure 2:**
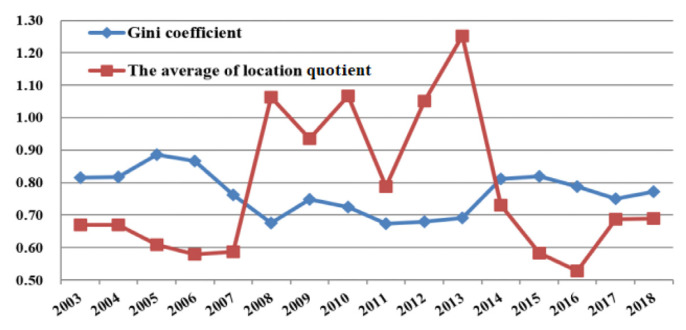
Gini coefficient and location quotient average trend chart venture capital in China from 2003 to 2018.

**Figure 3:**
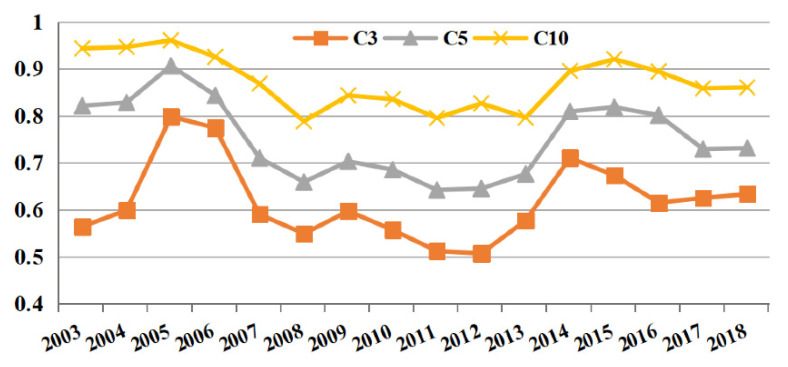
Concentration coefficient of venture capital in China from 2003 to 2018.

In order to reflect the spatiotemporal dynamic process of venture capital spatial agglomeration in China, this paper calculated the gini coefficient, location quotient index, and concentration coefficient of venture capital. This paper found the following results through analysis:

1. By analyzing the changes of venture capital investment scale gini coefficient, the study has shown that the agglomeration level of venture capital investment scale reached the maximum in 2005 (the gini coefficient value of 2005 was 0.886). It indicates that the spatial distribution of venture capital investment in China was unbalanced, the regional distribution gap was large, and the agglomeration trend was obvious. Subsequently, the agglomeration level decreased gradually until 2012 (the gini coefficient value of 2012 was 0.674). However, after 2013, the gini coefficient level gradually increased, which proved that the agglomeration imbalance of venture capital investment began to increase after 2013. (as shown in Figure 2).
2. The calculation results showed that the concentration coefficient of China’s venture capital was relatively high. As shown in the Formula (2) of concentration coefficient, C*_n_* measures the proportion of the top *n* provinces with the largest venture capital amount. Referring to previous research experience, *n* is generally taken as 3, 5, and 10. The concentration coefficient of the top three regions with the largest venture capital amount increased from 0.564 to 0.633 during the period from 2003 to 2018, while the concentration coefficient of the top five and top 10 regions with the largest venture capital amount decreased from 0.821 and 0.943 to 0.731 and 0.860 during the period from 2003 to 2018. From the perspective of time dimension, the concentration coefficients of the top 3, 5 and 10 regions with the largest proportion of venture capital amount all showed an upward trend first, then a downward trend. It indicates that the concentration degree of venture capital gradually weakened at first and gradually increased after 2013. (as shown in Figure 3).
3. Through the calculation of location quotient coefficient of the provinces, we found that the areas of high location quotient index were mainly concentrated in Beijing, Shanghai, Guangdong, and other regions. From 2008 to 2013, there were more areas with quotient coefficient greater than 1. It was proved that the level of some regions with high degree of venture capital agglomeration (Beijing, Shanghai, Guangzhou, etc.) decreased, while the location quotient coefficient of some other provinces increased correspondingly. This led to the sudden changes of this coefficient from 2008 to 2013. It is also proved that at this stage, the degree of concentration of venture capital in China decreased. However, the number of regions where the location quotient coefficient of venture capital was greater than 1 decreased after 2013, which proved that the uneven distribution of venture capital in China was further enhanced. Similarly, the average location quotient of the whole country also presented the same changing trend. (as shown in Figure 2).

Combining the trends of the three indicators, it was found that the gini coefficient, location quotient index, and concentration coefficient basically reflected the same trend of Chinese venture capital agglomeration (as shown in Table 2). Based on the three above indicators, we could divide the research period into three periods: (1) from 2003 to 2007, after a short period of rising, the degree of spatial agglomeration of venture capital space began to decline significantly; (2) from 2008 to 2012, the overall degree of spatial agglomeration of venture capital had little change and remained at a low level; (3) from 2013 to 2018, the degree of spatial agglomeration of venture capital gradually increased, and the degree of regional differentiation increased again. It can be seen that the spatial distribution of venture capital projects changed with regional policies, sources of funds, and industrial development.

## 4. Empirical Analysis

### 4.1. Model Setup

After testing, in the case of three spatial weight matrices, both LR and Wald test results rejected SDM at the significance level of 1%, which could be reduced to the spatial error model or the spatial lag model. In other words, we can conclude that the SDM cannot be simplified to the SLM or SEM. At the same time, following the principle that the greater the goodness of fit and likelihood function, the better the model will be, we selected the spatial fixed effect model. Therefore, the regression model of this paper was set as follows:

Based on the above theoretical hypothesis and the selection of variables, the linear model was firstly constructed. In view of the stationary data, logarithms of variables on both sides of the formula were taken, respectively. The model was set as follows:

(8) $$lnLQ_{VC}{}_{it}=\alpha +\beta_{1}lnEco_{it}+\beta_{2}lnTec_{it}+\beta_{3}lnFin_{it}+\beta_{4}lnCom_{it}+\beta_{5}lnSer_{it}\;+\;\beta_{6}lnEnt_{it}\;+\beta_{7}lnLab_{it}+\mu_{i}+\lambda_{i}+\varepsilon_{i}{}_{t}$$

In the formula, β is the regression parameter, *i* represents all provinces in the research areas, ε is the random error term. The explained variable *LQ_VC_* is the concentration degree of venture capital in the research region measured by the locational quotient coefficient. *Eco* stands for the factor of economic, *Tec* stands for the factor of science and technology, *Fin* stands for the factor of financial environment, *Com* and *Ser* stands for the factors of social environment, *Ent* and *Lab* stands for the factors of entrepreneurial environment. $\mu_{i}$ and $\lambda_{i}$ represent space effect and time effect, respectively. $\varepsilon_{it}$ represents the random disturbance term.

### 4.2. Results Analysis

Empirical research was carried out mainly by using Matlab and Geoda software platforms. Moran’s I was calculated by using the degree of concentration of venture capital of study areas in China from 2003 to 2018. The spatial weight matrix adopted the economic distance spatial matrix, and the results are shown in Table 3.

As can be seen from Table 3, Moran’s I from 2003 to 2018 were all positive, and all passed the 1% significance test, indicating that the spatial distribution of venture capital in China had shown obvious spatial autocorrelation since 2001. That is to say, the region with a high concentration of venture capital had a high level of venture capital investment in its surrounding areas, so the development of venture capital had a certain spatial dependence. From the perspective of numerical evolution, Moran’s I value presented a cyclical fluctuation characteristic. In 2006, Moran’s I was 0.069 at the minimum, while in 2012, Moran’s I was 0.453 at the maximum.

#### 4.2.1. Spatial Econometric Model Regression Results for National Samples

The estimation results and analysis of the spatial econometric model were as follows:

According to the spatial econometric model built above, the model was estimated and tested by using Matlab2017a software (MathWorks, Natick, MA, USA). Table 4 shows the estimation results of SDM based on the spatial adjacency matrix, the geographic distance matrix, the economic distance matrix, and the corresponding goodness of fit. Since R^2^ only reflects the goodness of fit of the regression, in order to eliminate the influence of the number of independent variables on R^2^, adjusted R^2^ was used in this paper. It can be seen from Table 4 that the goodness of fit of the OLS (Ordinary Least Squares) model was only 0.645, which was smaller than the 0.678, 0.680, or 0.681 of the spatial durbin model. Meanwhile, the value was significantly positive at the significance level of 1%, indicating that the spatial overflow effect existed in the explained variable, and the spatial econometric model was more appropriate. Compared with OLS, the accuracy of the SDM models was greatly improved. So, it was more reasonable to add spatial factors to analyze the effect of spatial agglomeration of venture capital. The estimated results show that the concentration of venture capital was not only affected by the local venture capital status, but also by the surrounding venture capital and related explanatory variables.

Under different spatial weights, its influence degree was also different. The spatial autocorrelation coefficient (ρ) was positive in the models and passed the significance test, indicating that the explained variable had an obvious spillover effect. It showed that the venture capital agglomeration in surrounding areas had a great impact on local venture capital investment. The spillover effect of based on the economic distance matrix (0.210) was generally larger than the spatial spillover effect based on the spatial adjacency matrix (0.201) and the geographic distance matrix (0.199), indicating that venture capital investment concentration had a relatively large correlation with the weight of economic distance, considering the level of regional economic development. China’s economic development has strong regional characteristics, and economic growth also shows the characteristics of “club convergence”. Meanwhile, by using the economic distance spatial weight matrix, the models will not break the links between nonadjacent provinces. In addition, venture capital attaches great importance to regional science and technology, finance, entrepreneurial atmosphere and other factors, and has a preference for site selection in areas with transparent information. Therefore, it is in line with the characteristics of venture capital industry that the spillover effect based on economic distance was greater than that based on spatial distance. Considering the goodness of fit and logarithmic likelihood, the estimation effect of SDM based on economic distance was better than other models.

Economic environment is the most important factor affecting the spatial agglomeration of venture capital. From the view of the estimated results, an estimated parameter of the economic development level was positive and passed the test of significance, so it showed that economic development level had great influence on venture capital agglomeration. At the same time, it was found that the estimated parameters of the spatial econometric model with spatial factors were generally smaller than OLS, which indicates that OLS estimates exaggerated the role of economic development level while ignoring spatial factors. In China, the regional economic development environment that attracts venture capital investment includes the regional economic development level, economic development strategy, economic system, market perfection, price level, and economic stability. Before investing, venture capitalists will first examine the economic development environment in the region, especially in the less developed inland regions. In the results of SDM, the estimated parameters of the economic development level factor were positive and very significant, indicating that the economic development level of the surrounding areas had a significant effect on the clustering of regional venture capital. Moreover, from the estimation results, the economic development level factor based on the economic distance matrix (1.341) was higher than that based on the geographical distance matrix (1.319) and the spatial adjacency matrix (1.257). It shows that the spatial distribution of venture capital was affected by economic spatial distance in order to obtain higher returns, and regions with a high economic development level can attract the inflow of venture capital. If a region lacks a stable economic development foundation and favorable economic development conditions, the investment risk of venture capital will be increased to some extent, and the attraction of the region to venture capital will be reduced accordingly. In SDM results, the estimated parameters of the spatial lag term of the economic development level were positive and relatively significant. It indicates that the economic development level of the surrounding areas had an obvious promoting effect on the spatial agglomeration of local venture capital, and the influence degree based on the economic distance matrix (0.481) was obviously higher than that of the geographical distance matrix (0.428). It indicates that in order to obtain more revenue, venture capital was affected by economic spatial distance, and areas with a high level of economic development can attract more venture capital inflows.

Scientific and technological environmental factor had a positive effect on the spatial clustering of venture capital, and most of them passed the significance test. Meanwhile, the estimation results of science and technology factors in the SDM model based on three spatial weight matrices (0.304, 0.305, 0.310) and in the OLS model (0.302) were basically consistent. It proved that this factor was not restricted by distance. It indicates that regional differences in scientific and technological innovation ability would expand the spatial clustering degree of venture capital and it was no longer limited by distance. Hot spots of venture capital such as Beijing and Shanghai drive their economic development by innovation, mainly relying on high-tech industries to drive the growth of the new economy. These places also have high efficiency in the transformation of scientific and technological achievements, which is conducive to the spatial agglomeration of venture capital activities.

Financial environment factor had a weak correlation and positive effect on the spatial agglomeration of venture capital, indicating that regional venture capital is largely affected by the economic development level, scientific and technological level, and the developed level of information infrastructure construction in each region. The influence of the amount of deposits and loans of financial institutions, and the degree of financial environment development on the regional distribution pattern of total venture capital was limited.

The two variables selected by social environmental factors, communication and network development level and social service level, were all positive in the estimated parameters of the model, and most of them passed the significance level test. The reason was that, in order to reduce its investment risk, venture capital is highly dependent on information, so its requirements on the development level of the regional communication network infrastructure will be increasingly high. At the same time, the development of regional venture capital requires a high level of facilities and services to match, so as to ensure the level of industry standardization and maturity of the whole market.

In the end, the two indexes selected for entrepreneurial environment were positive and negative, respectively, both with high levels of significance. Among them, the level of entrepreneurial activity was significantly positively correlated with spatial agglomeration of venture capital. Since venture capital is an effective incentive mechanism for entrepreneurial activities, venture capital is generally concentrated in regions with high levels of entrepreneurial activities. However, the rigidity index of the labor market had a weak negative correlation with the spatial agglomeration of venture capital, which proved that the rigid index of labor market inhibits venture capital activities.

As shown in Table 5, the direct and indirect effects (overflow effects) of variables based on three spatial weight matrices are listed. The factors of economic development level had significant direct effects and spillover effects (both passed the significance test of 10%), that is, the economic development level not only played a significant role in the spatial agglomeration of venture capital in this region, but also promoted the spatial agglomeration of venture capital in the surrounding areas. Moreover, the spillover effect based on the economic distance matrix (0.320) was obviously stronger than that based on the adjacent space matrix (0.125) and the geographic distance matrix (0.225). The direct effects and spillover effects of scientific and technological environmental factor, which had certain influence on the spatial agglomeration of venture capital. The direct effects of financial development level were weaker than the spillover effects in influencing the spatial agglomeration of venture capital. The direct effects of the entrepreneurial activity level factor and communication and network development level factor on the spatial agglomeration of venture capital were positive and significant, while the significance of the spillover effects level was poor. The direct effects of social services level on the spatial agglomeration of venture capital was large, and its spillover effects were obvious. The direct and indirect effects of labor market rigidity level factor on the spatial agglomeration of venture capital were negative, and the significance was weak.

#### 4.2.2. Regression Results of Different Time Periods

According to the above analysis, the spatial agglomeration degree of venture capital can be divided into three stages from the time dimension. Since the development stage of China’s economy and the factors affecting the spatial agglomeration of venture capital will vary with time, it is necessary to make a regression analysis of different time periods.

As shown in Table 6, by comparing the coefficients of each influence factor in the three stages, the following was found: Although the influence factor coefficients such as the level of scientific and technological innovation ability, the level of financial development, and the level of labor market rigidity have been improved to a certain extent compared with that of 2003–2007, the coefficients of economic development level, social service level, and entrepreneurial activity level in the period of 2008–2012 were significantly lower than those in the period of 2003–2007. It shows that the decrease of the spatial concentration of venture capital was significantly affected by the level of economic development, social service, and entrepreneurial activities during this period. The reason is that after the financial crisis in 2008, the operation of the real economy, the level of enterprise entrepreneurship, and the development of tertiary industry were gradually affected. Therefore, the economic environment that the venture capital industry depends on was damaged. Furthermore, the operation foundation and industrial chain of the venture capital industry were broken, and its financing level and exit channels were also seriously affected. These situations had a direct impact on the development of the venture capital industry in Beijing, Shanghai, and other places. The concentration degree of venture capital hot areas declined, which inhibited the concentration degree of venture capital industry in provinces. These situations had a direct impact on the development of the venture capital industry in Beijing and Shanghai. As a result, the concentration of venture capital hot areas decreased, which inhibited the concentration of venture capital industry in provinces.

At the same time, it was found that over time, factors affecting the spatial agglomeration of venture capital significantly changed in importance. From 2013 to 2018, it was found that the coefficients of the three indexes of economic development level, social services level, and labor market rigidity level were significantly larger than those of the previous stage, while the other influencing factors were relatively unchanged. It shows that after 2013, the rapid development of social economy and the supporting development of related service industries had significantly promoted the development and spatial agglomeration of venture capital industry in Beijing, Shanghai, and other regions.

#### 4.2.3. Regression Results of Eastern, Central and Western Region Samples

According to the economic development level and geographical position of each province, China’s mainland is divided into three regions: eastern region, central region, and western region. The eastern region includes Beijing, Tianjin, Hebei, Liaoning, Shanghai, Jiangsu, Zhejiang, Fujian, Shandong, Guangdong, Guangxi, and Hainan. The central region includes Shanxi, Inner Mongolia, Jilin, Heilongjiang, Anhui, Jiangxi, Henan, Hubei, and Hunan; The western region includes Guangxi, Chongqing, Sichuan, Guizhou, Yunnan, Shaanxi, Gansu, Qinghai, Ningxia, and Xinjiang. Because east, central, and west regions are divided by economic region, it is meaningful to make spatial econometric analysis according to economic distance weight matrix. Similarly, the spatial durbin model was used for regression, and the results are shown in Table 7.

By comparing the results in Table 7, it was found that there were certain differences in the regression coefficient and significance level of each influence factor in eastern and western regions. Among them, the significance level of each factor in the eastern region was the highest, while that in the central region was the lowest. It also can be seen from Table 7 that the values of the spatial autocorrelation coefficient (ρ) in the eastern, central, and western regions were all significantly positive (0.210, 0.168, and 0.251), indicating that there was a significant positive spatial spillover effect in the cluster of venture capital in the eastern, central, and western regions. In terms of the degree of spillover, the central region was the smallest, the western region was the highest, and the eastern region was in the middle. This may be because the western development strategy was put forward the earliest. Under the influence of the western development strategy, the policies, institutions, economic, social and cultural characteristics of western provinces are more similar, and the spillover effect of the degree of spatial agglomeration of venture capital is the largest. The strategy for the rise of central China was put forward last, and the development of the central region was the least coordinated, so the spatial spillover effect of venture capital was the lowest.

## 5. Conclusions and Implications

### 5.1. Conclusions

China’s venture capital industry has been developing in China for only 30 years and has just entered a stable stage of development. Therefore, it is challenging and urgent work to study it. By constructing a spatial econometric model of influencing factors of financial agglomeration, this paper reveals the internal formation mechanism of China’s venture capital spatial agglomeration.

The main conclusions and inspirations are as follows:

From the perspective of provincial scale, both venture capital institutions and venture capital projects showed significant agglomeration characteristics, which were very similar in spatial form, mainly concentrated in the first-tier cities and the eastern coastal areas. It shows that venture capital had local investment preference. At the same time, the phenomenon of spatial agglomeration of venture capital in China had spatial autocorrelation in section units, which forms strong spatial dependence and positive spatial spillover effect among provinces. This positive spatial spillover effect indicates that the development of venture capital in neighboring provinces can promote the improvement of venture capital in the region.
By constructing a spatial econometric model based on three different spatial weight matrices, it was found that there was a strong spatial dependence and positive spillover effects of space in venture capital agglomeration between the provinces in China. Considering the goodness of fit and logarithmic likelihood, the estimation effect of SDM based on economic distance was better than that of other models. The spatial distribution pattern of venture capital was mainly influenced by factors of economic environment, science and technology environment, financial environment, social environment, and entrepreneurial environment. First, economic environment is the most important factor affecting the spatial agglomeration of venture capital. Secondly, scientific and technological environmental factor had a positive effect on the spatial clustering of venture capital, and most of them passed the significance test. Thirdly, financial environment factor had a weak correlation and positive effect on the spatial agglomeration of venture capital. At the same time, the development of regional venture capital requires a high level of facilities and services to match, so as to ensure the level of industry standardization and maturity of the whole market. In the end, the two indexes selected for entrepreneurial environment were positive and negative, with a high level of significance.
Due to the degree of spatial agglomeration, venture capital can be divided into three stages in terms of time. Combined with the three indicators of Gini coefficient, location quotient index, and concentration coefficient, we can divide the period of research time into three stages: (1) from 2003 to 2007, (2) from 2008 to 2012, (3) from 2013 to 2018, the degree of spatial agglomeration of venture capital experienced a short rise, then a significant decline, and then a gradual increase. It can be found that the factors affecting spatial agglomeration of venture capital changed significantly with the passage of time.
According to the economic development level and geographical position of each province, the study area was divided into three regions: eastern region, central region, and western region. From the regression results of eastern, central, and western region samples, we can see that the degree of spillover effect was the lowest in the central region, the highest in the western region, and the middle in the eastern region.

### 5.2. Implications

Based on the empirical analysis of the measurement of spatial agglomeration characteristics of venture capital in China and its spatial spillover effect, combined with the reality of China’s industrial economic transformation, this paper puts forward the following policy recommendations:

(1) Firstly, this paper proposes to strengthen regional venture capital cooperation between neighboring provinces and regions, promote the free flow of venture capital between regions, so as to promote the optimal allocation of venture capital in a larger scope and promote the integration of the financial market.
(2) Secondly, the development of venture capital requires relevant economic environment and supporting industries, such as law firms, accounting firms, evaluation institutions, research institutions, etc. These institutions serve many venture capital funds and can better play to their professional advantages. At the same time, under the condition of insufficient overall scale, some regions are encouraged to guide the development of venture capital in policy. The support of venture capital should be focused on a region, and the development of a high-quality venture capital cluster should be cultivated. The government should support the development of the venture capital industry and cultivate high-quality venture capital clusters in different regions.
(3) Scientific and technological innovation ability, financial development, communication and network development, social services, and entrepreneurial activity level are the external environment of venture capital in the cluster area of venture capital. Therefore, the government should play a greater role in creating an institutional environment for the development of venture capital. The government can promote the development strategy and create a cultural and social atmosphere that encourages enterprises to innovate in science and technology. Only by advocating technological innovation as the core of competition, encouraging enterprise innovation and industrial cooperation, and ensuring the activity of competitive market, can the positive spillover effect of venture capital be brought into full play. By guiding the spillover mechanism of venture capital agglomeration, it can promote the driving effect of regional economic development and industrial transformation and upgrading.

This study focused on the spatial and temporal pattern and agglomeration characteristics of venture capital. By attempting to explore the evolution characteristics and influence mechanism of Chinese venture capital spatial agglomeration, this research extended the study of traditional financial subjects to the emerging financial subject of venture capital. Therefore, the main contribution of this project was expanding the research field of financial geography in China, enriching the research theory of financial geography in China, and providing references for the formulation of national and regional development policies. 

The spatial econometric model constructed in this paper to investigate the factors affecting the spatial agglomeration of venture capital can be extended and applied from multiple perspectives. At the same time, this paper also explored the influencing factors of spatial agglomeration of venture capital from the perspective of spatial-temporal characteristics and considered the geographical distance and economic distance in the selection of weights. However, in the independent variables, the variables selected in this paper were mainly considered. In addition, factors such as fixed asset investment level, number of financial industry employees, government intervention, and institutional environment can also be considered to conduct a more detailed and comprehensive investigation. This is what we need to improve and strengthen in future research.

## Figures and Tables

**Table 1:** Variable definition and description.

	Name of the Variable	Description of Variable	Specific Indicators
**Dependent Variable**	Degree of concentration of venture capital		Location quotient index of venture capital (*LQ_VC_*)
**Explaining Variable**	Factor of economic environment	Economic development level	Regional per capita GDP (*Eco*)
	Factor of science and technology environment	Scientific and technological innovation ability level	Number of patents (including invention patents, designs and utility models) (*Tec*)
	Factor of financial environment	Financial development level	Total deposits and loans of financial institutions at the end of the year (ten thousand RMB) (*Fin*)
	Factor of social environment	Communication and network development level	Regional per capita transportation and communication expenses (RMB/person) (*Com*)
		Social services level	Proportion of tertiary industry (%) (*Ser*)
	Factor of entrepreneurial environment	Entrepreneurial activity level	The number of private companies owned by people aged 15–60 in the region (*Ent*)
		Labor market rigidity level	The proportion of state-owned employment in the region (%) (*Lab*)

**Table 2:** Gini coefficient, location quotient, and concentration coefficient of venture capital in China.

Year	Gini Coefficient	C_3_	C_5_	C_10_	Location Quotient	
					The Number of Provinces (E > 1)	The Average of Location Quotient
2003	0.816	0.564	0.821	0.943	5	0.675
2004	0.816	0.599	0.828	0.946	5	0.669
2005	0.886	0.798	0.906	0.960	4	0.609
2006	0.867	0.774	0.843	0.925	3	0.579
2007	0.763	0.590	0.710	0.868	6	0.587
2008	0.676	0.549	0.659	0.788	11	1.063
2009	0.748	0.597	0.703	0.843	7	0.935
2010	0.724	0.557	0.685	0.835	8	1.068
2011	0.674	0.512	0.642	0.795	8	0.787
2012	0.679	0.507	0.645	0.826	8	1.051
2013	0.691	0.577	0.676	0.796	10	1.252
2014	0.811	0.710	0.809	0.895	3	0.730
2015	0.819	0.673	0.818	0.920	5	0.583
2016	0.788	0.615	0.801	0.894	5	0.527
2017	0.751	0.625	0.729	0.858	5	0.687
2018	0.772	0.633	0.731	0.860	6	0.690

**Table 3:** Moran’s I and its Z value of the degree of concentration of venture capital in the study areas.

Year	Moran’s I	Z Statistic	Year	Moran’s I	Z Statistic
2003	0.311	3.502	2011	0.298	3.652
2004	0.348	3.437	2012	0.453	4.355
2005	0.293	3.843	2013	0.248	2.973
2006	0.069	1.919	2014	0.190	2.854
2007	0.284	3.664	2015	0.337	3.738
2008	0.255	3.377	2016	0.383	4.364
2009	0.234	3.656	2017	0.250	3.005
2010	0.220	3.325	2018	0.310	3.871

**Table 4:** The regression results based on three spatial weight matrices and the spatial durbin model.

Variables	OLS	SDM		
		Adjacent Matrix	Geographical Distance Matrix	Economic Distance Matrix
*Eco*	1.152 ***	1.257 ***	1.319 ***	1.341 ***
	(5.822)	(5.693)	(6.133)	(5.413)
*Tec*	0.302 **	0.304 **	0. 305 **	0.310 **
	(2.034)	(2.126)	(2.143)	(2.596)
*Fin*	0.080 ***	0.046 *	0.046 **	0.043 ***
	(4.531)	(2.405)	(2.501)	(2.714)
*Com*	0.301 **	0.304 **	0.324 **	0.352 **
	(2.362)	(2.021)	(2.837)	(2.722)
*Ent*	0.213 **	0.387 **	0.337 **	0.690 ***
	(2.260)	(2.299)	(2.324)	(4.135)
*Ser*	1.009 **	1.771 ***	1.484 ***	1.980 ***
	(2.801)	(4.374)	(3.205)	(5.099)
*Lab*	−0.050 *	−0.063 *	−0.068 *	−0.088 *
	(−1.460)	(−1.575)	(−1.054)	(−1.247)
*W × Eco*		0.337	0.428 *	0.481 **
		(0.861)	(1.253)	(2.057)
*W × Tec*		0.554 **	0.595 *	0.590 *
		(2.473)	(1.741)	(1.587)
*W × Fin*		−0.504 *	−1.083	−1.373 *
		(−1.396)	(−0.816)	(−1.725)
*W × Com*		−0.228	−1.150	1.114 *
		(−0.481)	(−1.088)	(1.278)
*W × Ent*		−0.336	−0.632	−0.011
		(−1.079)	(−0.537)	(−0.021)
*W × Ser*		0.511	−3.806 *	−3.361 **
		(0.525)	(−1.086)	(−2.727)
*W × Lab*		−0.116	−0.401	−0.351 *
		(−0.824)	(−0.644)	(−1.153)
*ρ*		0.199 **	0.201 **	0.210 **
		(2.085)	(2.101)	(2.292)
*Adj-squared*	0.645	0.678	0.680	0.681
*Log-L*	225.81	−583.369	−581.796	−581.408

Note: ***, ** and * respectively mean passing significance test at the levels of 1%, 5%, and 10%.

**Table 5:** Direct and indirect effects of explanatory variables based on three spatial weight matrices.

Variables	Adjacent Matrix		Geographical Distance Matrix		Economic Distance Matrix	
	Direct Effect Coefficient	Indirect Effect Coefficient	Direct Effect Coefficient	Indirect Effect Coefficient	Direct Effect Coefficient	Indirect Effect Coefficient
*Eco*	1.271 ***	0.125 *	1.350 ***	0.225 **	1.329 ***	0.320 **
	(5.874)	(1.339)	(5.517)	(2.155)	(5.173)	(2.906)
*Tec*	0.131 **	0.015	0.308 **	0.019	0.351 *	0.022
	(2.354)	(0.122)	(2.011)	(0.147)	(1.301)	(0.671)
*Fin*	0.046 **	0.105 **	0.046 **	0.114 *	0.045 ***	0.106 **
	(2.409)	(2.541)	(2.449)	(1.674)	(4.275)	(2.192)
*Com*	0.108 *	−0.001	0.105 **	0.014	0.116 **	0.011
	(2.418)	(−0.039)	(2.206)	(0.026)	(2.504)	(0.322)
*Ent*	0.386 **	0.004	0.266 *	0.078	0.692 ***	0.066 *
	(2.262)	(0.142)	(1.472)	(0.307)	(4.170)	(1.901)
*Ser*	1.769 ***	0.717 **	1.529 ***	0.788 **	2.017 ***	0.995 **
	(4.225)	(2.137)	(3.301)	(2.208)	(5.334)	(2.904)
*Lab*	−0.061 *	−0.001	−0.073	−0.019	−0.086 *	−0.008
	(−1.639)	(−0.053)	(−1.008)	(−0.135)	(−1.206)	(−0.610)

Note: ***, ** and * respectively mean passing significance test at the levels of 1%, 5%, and 10%.

**Table 6:** Regression results of different time periods in 2003–2007, 2008–2012, and 2013–2018.

Variables	2003–2007		2008–2012		2013–2018	
	Coefficient	Coefficient (W × Variable)	Coefficient	Coefficient (W × Variable)	Coefficient	Coefficient (W × Variable)
*Eco*	1.294 ***	0.648 **	0.625	0.989 **	1.952 ***	−0.680
	(4.189)	(1.809)	(1.355)	(2.686)	(4.184)	(−0.638)
*Tec*	0.225 *	−1.701 **	0.124 *	2.040 ***	0.311 ***	1.008**
	(1.877)	(−1.713)	(1.767)	(3.515)	(2.578)	(2.201)
*Fin*	0.027 **	−2.434 *	0.009 **	−2.005	0.096 **	−0.440
	(2.669)	(−1.410)	(2.253)	(−1.448)	(2.424)	(−0.516)
*Com*	0.007 **	1.322 *	0.033 **	2.017 *	0.033 **	1.059 *
	(−2.013)	(1.602)	(2.092)	(1.499)	(2.102)	(1.856)
*Ent*	1.409 ***	3.911 **	0.416 *	−1.549 *	0.539 ***	−0.969 *
	(3.541)	(2.503)	(1.436)	(−1.714)	(3.339)	(−1.725)
*Ser*	2.068 **	0.319	1.318 **	−7.187 ***	2.599 ***	−4.933 ***
	(2.141)	(0.106)	(2.308)	(−3.331)	(5.586)	(−2.785)
*Lab*	−0.845 *	3.115	−0.602 *	−2.162 *	−0.030 *	−0.112
	(−1.963)	(0.863)	(−1.388)	(−1.316)	(−1.586)	(−0.475)
ρ	0.247 **		0.134 *		0.287 ***	
	(2.326)		(1.918)		(3.604)	
observation	155		155		186	
Log-likelihood	−234.070		−180.523		−149.888	
Adj-Squared	0.662		0.687		0.827	

Note: ***, ** and * respectively mean passing significance test at the levels of 1%, 5%, and 10%.

**Table 7:** Regression results of eastern, central, and western region samples.

Variables	East Region		Central Region		West Region	
	Coefficient	Coefficient (W × Variable)	Coefficient	Coefficient (W × Variable)	Coefficient	Coefficient (W × Variable)
*Eco*	0.700 ***	0.473	0.738 *	−1.610	1.578 ***	7.576 ***
	(2.870)	(0.405)	(1.352)	(−0.897)	(3.204)	(3.804)
*Tec*	0.345 **	−0.390	0.347 *	0.110	0.036 *	−1.188
	(1.955)	(−0.846)	(1.781)	(0.232)	(1.466)	(−1.357)
*Fin*	0.395 *	−0.909	0.541	−1.990 *	0.259	2.710 *
	(1.352)	(−0.949)	(0.760)	(−1.444)	(0.626)	(1.653)
*Com*	1.023 ***	2.580 **	0.265	0.489	0.469 *	4.644 ***
	(2.661)	(2.135)	(0.287)	(0.213)	(1.837)	(2.844)
*Ent*	0.772 ***	1.084 *	1.209 **	1.007	0.736 **	0.516
	(3.391)	(1.625)	(2.391)	(1.256)	(2.371)	(0.523)
*Ser*	2.306 **	−2.066	−0.474 *	−7.621 *	0.423 *	9.594 **
	(2.227)	(−1.095)	(−2.009)	(−2.057)	(2.024)	(2.348)
*Lab*	−0.548 *	−1.083	0.092*	−1.802	−0.018 *	0.268
	(−1.896)	(−0.741)	(1.830)	(−0.550)	(−1.212)	(0.862)
ρ	0.210 **		0.168 *		0.251 ***	
	(2.605)		(1.750)		(3.512)	
observation	168		126		140	
Log-likelihood	−173.974		−144.577		−184.960	
Adj-Squared	0.809		0.330		0.518	

Note: ***, ** and * respectively mean passing significance test at the levels of 1%, 5%, and 10%.

## Data Availability

The data presented in this study are available on request from the corresponding author.
